# Morphometric features of drug-resistant essential tremor and recovery after stereotactic radiosurgical thalamotomy

**DOI:** 10.1162/netn_a_00253

**Published:** 2022-07-01

**Authors:** Thomas A. W. Bolton, Dimitri Van De Ville, Jean Régis, Tatiana Witjas, Nadine Girard, Marc Levivier, Constantin Tuleasca

**Affiliations:** Department of Clinical Neurosciences, Neurosurgery Service and Gamma Knife Center, Centre Hospitalier Universitaire Vaudois (CHUV), Lausanne, Switzerland; Connectomics Laboratory, Department of Radiology, Centre Hospitalier Universitaire Vaudois (CHUV), Lausanne, Switzerland; Institute of Bioengineering, Ecole Polytechnique Fédérale de Lausanne (EPFL), Lausanne, Switzerland; Department of Radiology and Medical Informatics, University of Geneva (UNIGE), Geneva, Switzerland; Stereotactic and Functional Neurosurgery Service and Gamma Knife Unit, Assistance Publique-Hôpitaux de Marseille, Centre Hospitalier Universitaire de la Timone, Marseille, France; Neurology Department, Assistance Publique-Hôpitaux de Marseille, Centre Hospitalier Universitaire de la Timone, Marseille, France; Department of Diagnostic and Interventional Neuroradiology, Centre de Résonance Magnétique Biologique et Médicale, Assistance Publique-Hôpitaux de Marseille, Centre Hospitalier Universitaire de la Timone, Marseille, France; Faculty of Biology and Medicine (FBM), University of Lausanne (UNIL), Lausanne, Switzerland; Signal Processing Laboratory (LTS 5), Ecole Polytechnique Fédérale de Lausanne (EPFL), Lausanne, Switzerland

**Keywords:** Essential tremor, Radiosurgery, Thalamotomy, Ventro-intermediate nucleus, Structural covariance analysis, Morphometry, Cortical thickness, Surface area, Mean curvature

## Abstract

Essential tremor (ET) is the most common movement disorder. Its neural underpinnings remain unclear. Here, we quantified structural covariance between cortical thickness (CT), surface area (SA), and mean curvature (MC) estimates in patients with ET before and 1 year after ventro-intermediate nucleus stereotactic radiosurgical thalamotomy, and contrasted the observed patterns with those from matched healthy controls. For SA, complex rearrangements within a network of motion-related brain areas characterized patients with ET. This was complemented by MC alterations revolving around the left middle temporal cortex and the disappearance of positive-valued covariance across both modalities in the right fusiform gyrus. Recovery following thalamotomy involved MC readjustments in frontal brain centers, the amygdala, and the insula, capturing nonmotor characteristics of the disease. The appearance of negative-valued CT covariance between the left parahippocampal gyrus and hippocampus was another recovery mechanism involving high-level visual areas. This was complemented by the appearance of negative-valued CT/MC covariance, and positive-valued SA/MC covariance, in the right inferior temporal cortex and bilateral fusiform gyrus. Our results demonstrate that different morphometric properties provide complementary information to understand ET, and that their statistical cross-dependences are also valuable. They pinpoint several anatomical features of the disease and highlight routes of recovery following thalamotomy.

## Author Summary

Doubts remain regarding the anatomical alterations underlying essential tremor, partly owing to heterogeneity in symptoms’ severity and response to medication. Here, we studied drug-resistant patients clinically assessed and imaged before as well as 1 year after stereotactic radiosurgical thalamotomy, which significantly lowered tremor intensity. We extracted morphometric estimates of volume (subcortex and cerebellum), cortical thickness, surface area, and mean curvature (cortex), and quantified cross-regional statistical dependences across subjects (i.e., structural covariance or SC) for each measure, as well as cross-measure relationships for each region. Compared to matched healthy controls, patients showed altered surface area structural covariance within motion-related areas. Thalamotomy modulated mean curvature SC in frontal and subcortical centers. In both comparisons, SC and cross-measure relationship differences were also observed in visual areas.

## INTRODUCTION

Essential tremor (ET) is the most common movement disorder, affecting up to 1% of the population and 5% of individuals above 65 years of age (Louis & Ferreira, 2010). The main diagnosis is clinical. Patients present with postural and kinetic tremor of the upper limbs (hands and arms), sometimes completed by further head, legs, or voice tremor (Chunling & Zheng, 2016). In addition to motor symptoms, it has become increasingly clear that there are other possible concomitant manifestations of the disease, including cognitive deficits, psychiatric disorders, or sensory deficiencies (Chandran & Pal, 2012; Jhunjhunwala & Pal, 2014; Louis, 2016).

The exact etiology of ET remains largely elusive. While a genetic origin is unequivocal, as more than 50% of individuals have a positive family history, no ET-specific gene mutation has so far been reliably identified, and genome-wide association studies have only enabled researchers to pinpoint few disease-causing genetically complex variants (Kuhlenbäumer et al., 2014; Siokas et al., 2020; Tio & Tan, 2016). Candidate genes are involved in a wide array of functions, as they may code for ion channels, partake in neuroregeneration, or contribute to axonal myelination (Deng et al., 2019). In addition, environmental and epigenetic influences are also hypothesized (Hopfner & Helmich, 2018).

This complex etiology translates to a pathophysiology that remains to be fully elucidated. While the key culprit brain regions are acknowledged to lie within the cerebello-dentato-thalamo-cortical network (commonly known as the *tremor network*), including the cerebellum—believed to be the cornerstone of ET by some (Benito-León & Labiano-Fontcuberta, 2016; Ibrahim et al., 2021)—thalamus, motor thalamus (ventro-intermediate nucleus, Vim), and motor cortex (Hallett, 2014; Sharifi et al., 2014), the exact underlying mechanisms remain debated. Some authors have suggested that ET may result from abnormal neuronal oscillations within the tremor network (Deuschl & Elble, 2009), while others consider it as a neurodegenerative disorder with progressive cell loss (Benito-León, 2014). A GABAergic dysfunction has also been evoked (Gironell, 2014). These theories are not mutually exclusive and can all be justified by convincing evidence, but their diversity shows that gathering a complete picture of ET remains an ongoing endeavor.

Many have used magnetic resonance imaging (MRI) to try and pinpoint the alterations in brain anatomy caused by ET. With voxel-based brain morphometry (VBM), decreases in gray matter (GM) density were observed in the cerebellum, frontal and parietal cortices, and insula (Benito-León et al., 2009). Atrophy of the cerebellum and the frontal, occipital, middle temporal, and superior parietal cortices was also put forward (Bagepally et al., 2012), while other works pinpointed atrophy of the cerebellum (Quattrone et al., 2008) and of the left temporal pole and occipital cortex (Tuleasca et al., 2017). The use of automated segmentation labeling instead of VBM confirmed cerebellar atrophy in ET (Cerasa et al., 2009), while also revealing lower thalamus, hippocampus,and ventral diencephalon volume, and larger right caudate, pallidum, amygdala, and bilateral putamen and nucleus accumbens volume (Pietracupa et al., 2019; Prasad et al., 2019).

At the level of cortical morphometric properties, Benito-León and colleagues reported that more severe tremor correlated with atrophy in medial orbitofrontal and lingual cortices, the supramarginal gyrus, paracentral lobule, and in reduced thalamus and amygdala areas (Benito-León et al., 2019). In another study, patients who did not respond to propranolol (a widely prescribed antitremor drug) showed diminished left orbitofrontal and right temporal CT (cortical thickness) compared to responders (Chung et al., 2013). Serrano et al. (2017) also found that the standard deviation of CT in the right inferior parietal and fusiform areas plays a key role in distinguishing between ET patients and healthy controls.

For such patients with ET who do not respond to commonly prescribed medication, several surgical options are available (Elble et al., 2018; Picillo & Fasano, 2016). While the standard intervention remains deep-brain stimulation (Benabid et al., 1996), during the past 20 years, minimally invasive stereotactic radiosurgery (SRS, particularly Gamma Knife, GK) of the Vim of the thalamus has also been proofed as safe and effective, in particular for patients with contraindication for open surgery or as a patient’s choice (Elaimy et al., 2010; Tuleasca, Najdenovska, et al., 2018). To date, the impacts of such an intervention (or existing alternatives) have primarily been studied through resting-state functional MRI (Jang et al., 2016; Park et al., 2017; Tuleasca, Regis, et al., 2018), but structural investigations at the level of anatomical brain properties remain lacking.

Here, we focus on drug-resistant patients who underwent unilateral GK radiosurgery of the left Vim for right dominant ET. Our primary aims were to (1) refine our understanding of ET before thalamotomy, by comparing our cohort of ET patients in the absence of medication to matched healthy controls (HCs), and (2) gain insight into potential brain plasticity mechanisms triggered by GK thalamotomy, by comparing the pre- and posttherapeutic (1 year after intervention) states to account for delayed clinical and radiological effects.

In order to achieve this, we quantified morphometric brain attributes. In addition to cerebellar and subcortical volume, we also extracted three regional cortical morphometric features: CT, SA (surface area) and mean curvature (MC). Several reasons motivated this multimeasure analysis: first, SA and MC have not yet been investigated in the context of ET. Second, each measure encodes, to an extent, unique information: for instance, in healthy subjects, they yield distinct cortical asymmetry maps (Chiarello et al., 2016). Third, they are genetically and phenotypically complementary (Sanabria-Diaz et al., 2010; Yang et al., 2016), which renders a joint analysis interesting as their interactions may be altered in ET. Fourth, each measure is modulated differently by environmental factors, such as urban upbringing (Besteher et al., 2017) or maltreatment (Kelly et al., 2013); thus, it is reasonable to expect that ET may also exert distinct impacts on them.

More specifically, we sought to complement previous morphometric works by leveraging structural covariance analysis (SCA) to study cross-regional dependences. In SCA—see Alexander-Bloch et al. (2013) and Evans (2013) for reviews—one is interested in the extent to which a measure of interest (e.g., CT) correlates across subjects in a pair of regions. Such patterns of covariance are a characteristic attribute of the human cortex (Mechelli et al., 2005), are genetically regulated (Morgan et al., 2019; Romero-Garcia et al., 2018; Schmitt et al., 2008), and partly reflect structural connectivity (Yee et al., 2018). Their relevance is further corroborated by the fact that the foci of atrophy in many brain disorders largely overlap with covariance patterns found when using the most atrophied locations as seeds in healthy subjects (Seeley et al., 2009). SCA has been a fruitful analytical approach to better comprehend the healthy and diseased brain (Bassett et al., 2008; Bernhardt et al., 2011; Bethlehem et al., 2017; Chen et al., 2008; He et al., 2008; Khundrakpam et al., 2017). To our knowledge, only one recent study has considered SCA in the context of ET (Yang et al., 2021), in which the authors quantified the similarity of GM profiles across regions to assess covariance and studied a cohort of drug-naïve ET patients. With this work, we seek to instead leverage SCA to study drug-resistant patients, and how they may improve following GK thalamotomy.

## MATERIALS AND METHODS

### Participants

We considered uniform structural MRI data from 34 ET patients (both prethalamotomy and 1 year later) scanned on the same 3T MR machine, and 29 HCs. All patients were right-handed and presented with drug-resistant right-dominant tremor. All underwent left Vim thalamotomy by GK.

The Timone University Hospital Ethical Committee (ID-RCB: 2017-A01249-44) granted formal approval for this study (including by the Ethics Committee at national level, CNIL-MR-03), and individual consent was also obtained from all subjects. Patients were neurologically evaluated and referred by T.W., a neurologist specialized in movement disorders. All patients had a clear diagnosis of ET and showed no other structural abnormalities on pretherapeutic 3T MRI. Demographic characteristics of the ET patients and HC subjects can be found in Table 1, where it can be seen that the groups were matched for both age and gender.

**Table 1.** Demographic and clinical details of the subjects

Variable	HC	ET_pre_	ET_post_	Drop (points)	Drop (%)	*N*_missing_	*P* value
*N*	29	34	34	n.a.	n.a.	n.a.	n.a.
Age (years)	69.93 ± 7.14 [59, 69, 83]	70.06 ± 9.12 [49, 72, 83]	n.a.	n.a.	n.a.	n.a.	*t*_66_ = −0.06, *p* = 0.95
Gender (M:F)	12:17	17:17	17:17	n.a.	n.a.	n.a.	n.a.
ADL	n.a.	29.59 ± 11.39 [13, 28.5, 49]	6.03 ± 11.26 [0, 1, 41]	−23.56 ± 12.35 [−48, −24.5, 2]	82.83 ± 29.64 [0, 96.75, 100]	0/0	***t*_66_ = 8.57, *p* = 2.48 · 10^−12^**
HEAD	n.a.	1 ± 0.85 [0, 1, 2]	0.56 ± 0.75 [0, 0, 3]	−0.39 ± 0.83 [−2, 0, 1]	n.a.	0/1	***t*_65_ = 2.16, *p* = 0.035**
QUEST	n.a.	45.46 ± 16.4 [12, 41.5, 80]	23.16 ± 16.57 [1, 26, 57]	−24.79 ± 13.21 [−47, −25, −2]	n.a.	8/9	***t*_43_ = 15.37, *p* = 4.47 · 10^−19^**
TSTH	n.a.	20.41 ± 5.53 [8, 20.5, 30]	6.26 ± 7.71 [0, 3, 27]	−14.15 ± 6.6 [−26, −14.5, 1]	72.73 ± 29.19 [0, 86.05, 100]	0/0	***t*_66_ = 8.69, *p* = 1.52 · 10^−12^**
Lesion volume (ml)	n.a.	0.12 ± 0.13 [0.002, 0.076, 0.6]	n.a.	n.a.	n.a.	n.a.	n.a.
Time to tremor arrest (days)	n.a.	n.a.	127.56 ± 81.38 [15, 120, 300]	n.a.	n.a.	2	n.a.
Symptoms duration (years)	n.a.	35.53 ± 18.28 [5, 33, 61]	n.a.	n.a.	n.a.	n.a.	n.a.

*Note*. For healthy controls (HCs), patients before (ET_pre_) and after thalamotomy (ET_post_), values are reported as mean ± standard deviation, with minimum, median, and maximum into squared brackets. Some clinical scores could not be collected in a few occasions (*N*_missing_), in which case the associated subjects were excluded from statistical computations. Significant statistical comparisons are highlighted in **bold**. M, male; F, female.

Several measures were used to clinically evaluate ET patients, and their potential recovery after thalamotomy: Activities of Daily Living from the survey designed by Bain and colleagues (Bain et al., 1993), Tremor Score on Treated Hand (TSTH) from the Fahn-Tolosa-Marín rating scale (Fahn et al., 1993), head tremor (Tremor Research Group Essential Tremor Rating Assessment, from 0 to 3), and Quality of Life in Essential Tremor (QUEST; Tröster et al., 2005). Clinical data is summarized in Table 1, where a significant improvement in clinical tremor scores can be observed across all the quantified measures upon thalamotomy. In what follows, we will focus on TSTH values before the intervention (to quantify the extent of tremor) and on the percentage change in TSTH from before to after the intervention (to address the extent of recovery).

Importantly, SCA is not compatible with the subject-wise investigation of these scores: indeed, only one measure of covariance is generated per group (e.g., posttherapeutic ET patients). Thus, one cannot conduct classical correlation analyses between morphometric features and clinical scores. In order to nonetheless account for the fact that different patients recovered to distinct extents, we devised a strategy to gauge a possible link between the extracted SC features and tremor symptoms or postinterventional recovery (see Link to Clinical Scores section).

### Imaging

The imaging data was acquired on a head-only 3T machine (SIEMENS SKYRA, Munich, Germany, 32-channel receive-only phase-array head coil). Native T1-weighted images were acquired before and 1 year after thalamotomy with the following parameters: TR/TE = 2 300/2.98 ms, isotropic voxels of 1 mm^3^, 160 slices.

As medication was frequently ineffective, most patients no longer received treatment at the time of thalamotomy. Scanning was performed in a drug-naïve state (drugs having been stopped at least 3 days beforehand).

### Stereotactic Radiosurgical Procedure

GK thalamotomy was performed using Leksell Gamma Knife (Elekta Instruments, AB, Sweden) between September 2014 and April 2016, always at the Centre Hospitalier Universitaire de la Timone in Marseille, by the same neurosurgeon (J.R.). To avoid artifacts, diffusion tensor imaging data was first acquired without the frame, and then co-registered with the therapeutic stereotactic images. The Leksell coordinate G frame (Elekta Instruments, AB, Sweden) was always applied under local anesthesia on the day of the GK thalamotomy. After positioning the frame, patients underwent both stereotactic CT and MRI.

Landmarks of interest, including the anterior and posterior commissures, were identified on an MR scan (particularly on T2 CISS/FIESTA sequence, Siemens). Uniform indirect targeting was performed using the Guiot diagram (Tuleasca, Regis, et al., 2018), placed 2.5 mm above the anterior-posterior commissure line, and 11 mm lateral to the wall of the third ventricle. A single 4-mm isocenter was always used, and a maximum prescription dose of 130 Gy at the 100% isodose line was uniformly prescribed (Tuleasca, Regis, et al., 2018).

### Computation of Morphometric Properties

The FreeSurfer software (Fischl, 2012) was used to extract three morphometric measures of interest from structural MR images for a set of *P*_cort_ = 68 cortical regions: CT, SA, and MC. We selected these because they have been widely used and acknowledged as relevant indicators of brain geometry; see, for example, Harnett et al. (2020), Raznahan et al. (2011), and Yang et al. (2013). We hoped to find possibly distinct relationships with ET and, in addition, considered to also explicitly quantify the interplay across properties (see Cross-Measure Analysis section).

Briefly, after linear registration to MNI space and bias field removal, the image at hand is skull-stripped (Ségonne et al., 2004), and voxels are classified as belonging to white matter or to another tissue category based on their intensity and direct neighborhood. Hemispheres are separated, the cerebellum and subcortex are removed, and the interface between the white and gray matter is located. From there, the pial surface is also tiled, and local estimates of CT, SA, and MC can be extracted (Fischl & Dale, 2000). Further details about these steps of the methodology can be found in Dale et al. (1999) and Fischl et al. (1999). Then, local voxel-wise measurements are converted into *P*_cort_ regional values per morphometric measure, using the Desikan-Killiany atlas (Desikan et al., 2006).

In addition to the *P*_cort_ = 68 cortical brain regions, we also extracted regional volume for *P*_noncort_ = 19 noncortical areas, including the cerebellum and subcortical nuclei. Supporting Information Table S1 summarizes the considered regions.

Of note, noncortical measures quantify regional volume instead of the three morphometric properties at hand. However, because we only assess cross-regional correlations, the distinct ranges of values do not impact our analyses. Thus, similarly for CT, SA, and MC, the *P*_noncort_ noncortical volume values were appended to the *P*_cort_ cortical estimates, resulting in a *P*-dimensional vector for each subject, with P = 87. Note that there is no redundancy in our analyses, since the correlational relationships between noncortical volume and a given morphometric measure may differ from those with another.

These measurements were eventually linearly regressed out for age, gender, and total gray matter volume, separately within each group. The obtained residuals were used for all subsequent analyses.

### Edge-Wise Analysis

The process described below was identically conducted for each morphometric measure of interest. In total, there are *P*(*P* − 1)/2 = 3,741 cross-regional edges available. Separately for the HC, prethalamotomy (abbreviated ET_pre_ from there onwards) and postthalamotomy (abbreviated ET_post_) data, Pearson’s correlation coefficient *R* was computed for a given edge. A positive/negative value means that when the measure in the first region is larger in one subject, it tends to be larger/lower in the second region. The coefficient of determination *R*^2^ denotes the associated percentage of explained variance.

We first attempted to exclude the edges that only reflect noise (i.e., for which the explained variance is minimal). Noisy edges were defined as those for which, across the HC, ET_pre_, and ET_post_ cases, the coefficient of determination was always lower than 0.2 (i.e., less than 20% explained variance). Formally, this amounts to *R*^2^ < 0.2 or |*R*| < 0.4472. We selected this threshold value as it is regarded as a moderate-to-high correlation across scientific subfields (Akoglu, 2018). In the specific context of SCA, recent work has also considered lower/larger values as low/high correlations and shown that fluctuations due to acquisition or preprocessing settings span a lower range of *R* = 0.01 to 0.1 (Carmon et al., 2020).

Our thresholding step excluded 2,206 edges for CT (amounting to 41.03% of edges kept), 2,990 for SA (20.07%) and 1,408 for MC (62.36%). Note that in line with the above criterion, when interpreting our results, we also consider a structural covariance negligible if *R*^2^ < 0.2.

On the remaining edges, to assess the differences between HC subjects and ET patients, we computed the difference HC-ET_pre_ for each edge. Similarly, to investigate the effects of thalamotomy, we computed the difference ET_post_-ET_pre_.

For statistical assessment, these differences must be compared to an appropriate null distribution. To do so, we resorted to nonparametric permutation testing, by recomputing covariance relationships after having randomly shuffled subjects across groups. In total, 300,000 null realizations were generated in each case, and false discovery rate (FDR)-corrected *p* values were obtained and analyzed.

### Link to Clinical Scores

SC estimates are obtained on full populations (e.g., the whole ET_pre_ set of subjects) and thus do not enable direct correlational analysis with the severity of clinical symptoms or the extent of recovery upon intervention. It is thus impossible, with these results alone, to know whether the most impaired subjects (or the ones who recovered the most) more strongly drove a given group difference.

In order to circumvent this limitation, we devised an analytical strategy in which all the significant group differences are recomputed upon the exclusion of a selected number of data points ranging from 1 to 10. For the HC-ET_pre_ case, we excluded either the *least* impaired subjects in terms of baseline TSTH (yielding an estimate SC_1_(*r*), with *r* the number of removed subjects), or the *most* impaired ones (SC_2_(*r*)). If the most impaired subjects cause the HC-ET_pre_ group difference, we would expect the first case to only slightly alter the results; conversely, the second case should yield a greater extent of change.

We computed the difference |SC_2_(*r*)| − |SC_1_(*r*)|, and took its average μ_SC_, and the regression coefficient of a linear model *β*_SC_ (*y* = *β*_SC_*r* + *c*, with *y* = |SC2(*r*)| − |SC1(*r*)|, *r* the number of subjects removed and *c* as constant) as summarizing metrics. A positive μ_SC_ value means that on the whole, the assessed group difference is larger when focusing on the most impaired as opposed to the least impaired subjects. A positive *β*_SC_ value means that as more subjects are removed in both cases, the group difference increases. We considered the connections for which both quantities were positive valued as showing an association with the extent of tremor, as quantified by baseline TSTH.

The same strategy was also applied to the ET_post_-ET_pre_ case, where the percentage of TSTH improvement was used as clinical score instead. In this case, positive-valued μ_SC_ and *β*_SC_ mean that a focus on the subjects that recover the most following the intervention (by excluding the 1 to 10 worst recoverers), as opposed to those that recover the least (by excluding the 1 to 10 best recoverers), magnifies the group difference. Such connections were considered as showing an association with the extent of postinterventional recovery, as quantified by the percentage of TSTH improvement.

### Cross-Measure Analysis

We were also interested in studying the covariance *across structural properties* within a region (for example, if an area is thicker in a subject, does it also tend to exhibit a greater surface area?). For this purpose, for each pair of morphometric measures and each cortical brain region, we computed the covariance across subjects. We then submitted the outputs to the same statistical pipeline as above for both the HC-ET_pre_ and the ET_post_-ET_pre_ cases to yield FDR-corrected *p* values.

Note that in the case of this analysis, we only perform *P*_cort_ = 68 assessments (one per cortical area); as we do not consider *cross-regional* (but rather *cross-measure*) relationships, the thresholding strategy described in the Edge-Wise Analysis section is not required.

### Availability of the Data and Scripts

All the analytical steps described above were performed with custom scripts and MATLAB2014b (MathWorks, Natick, USA). Color maps for plotting were generated with the *cbrewer* toolbox (https://www.mathworks.com/matlabcentral/fileexchange/34087-cbrewer-colorbrewer-schemes-for-matlab). All the scripts used in this work are freely available at https://github.com/TiBiUan/SCA_EdgeWise.git.

Data sharing is not applicable to this article as no new data were created or analyzed in this study.

## RESULTS

### Edge-Wise Analysis

**Figure 1.**
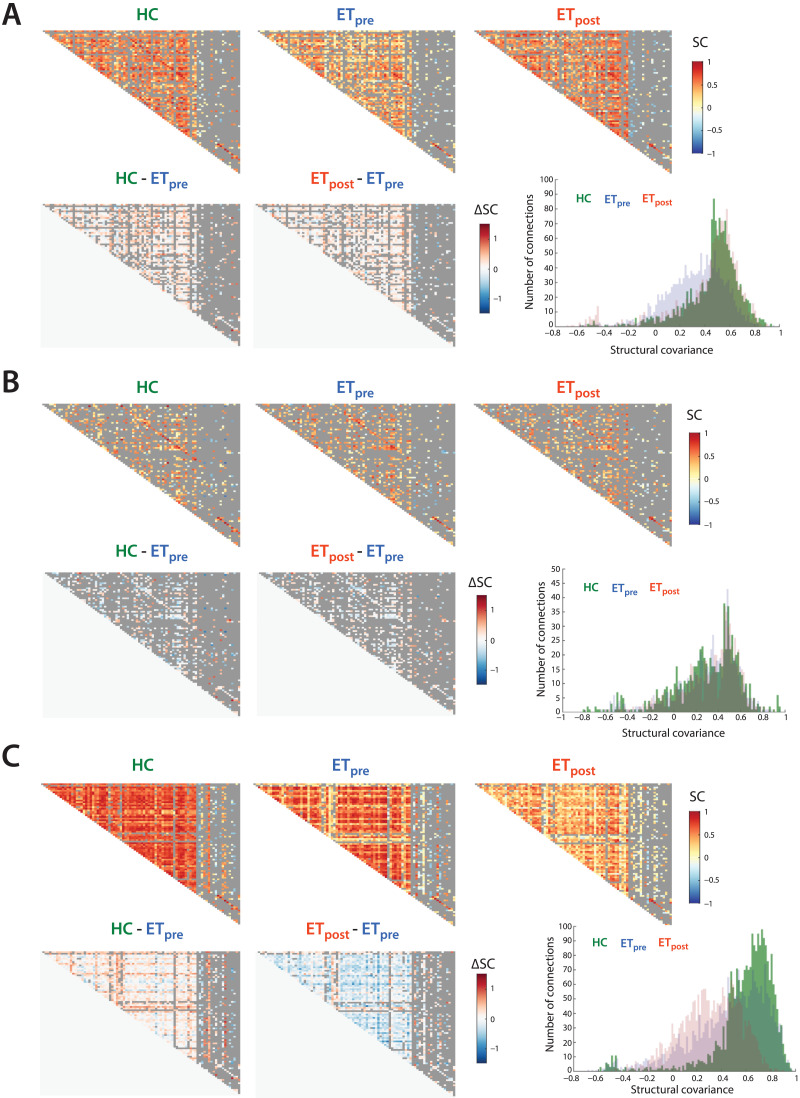
Structural covariance patterns across groups and modalities. For cortical thickness (A), surface area (B), and mean curvature (C), structural covariance matrices for the HC, ET_pre_, and ET_post_ groups (top row, from left to right), associated HC-ET_pre_ and ET_post_-ET_pre_ group differences (bottom row, left and middle panels), and summarizing histogram (bottom row, right panel). Discarded connections are depicted in gray.

Structural covariance matrices and SC value distributions for all three groups, as well as associated HC-ET_pre_ and ET_post_-ET_pre_ group differences, are shown in Figure 1. For CT (Figure 1A), structural covariance was overall lower in ET_pre_ subjects compared to the other two groups, as reflected by more SC values between −0.2 and 0.4 in the histogram. A few connections from the ET_post_ group also showcased particularly negative structural covariance (between −0.6 and −0.4). This was quantitatively confirmed by respective average HC, ET_pre_, and ET_post_ SC values of 0.25 ± 0.28, 0.15 ± 0.26, and 0.19 ± 0.32, and significant associated ET_pre_-HC (Wilcoxon rank sum test’s *z* = −15.56, *p* < 0.001), ET_post_-ET_pre_ (*z* = 4.8, *p* < 0.001) and ET_post_-HC (*z* = −8.61, *p* < 0.001) group differences.

**Figure 2.**
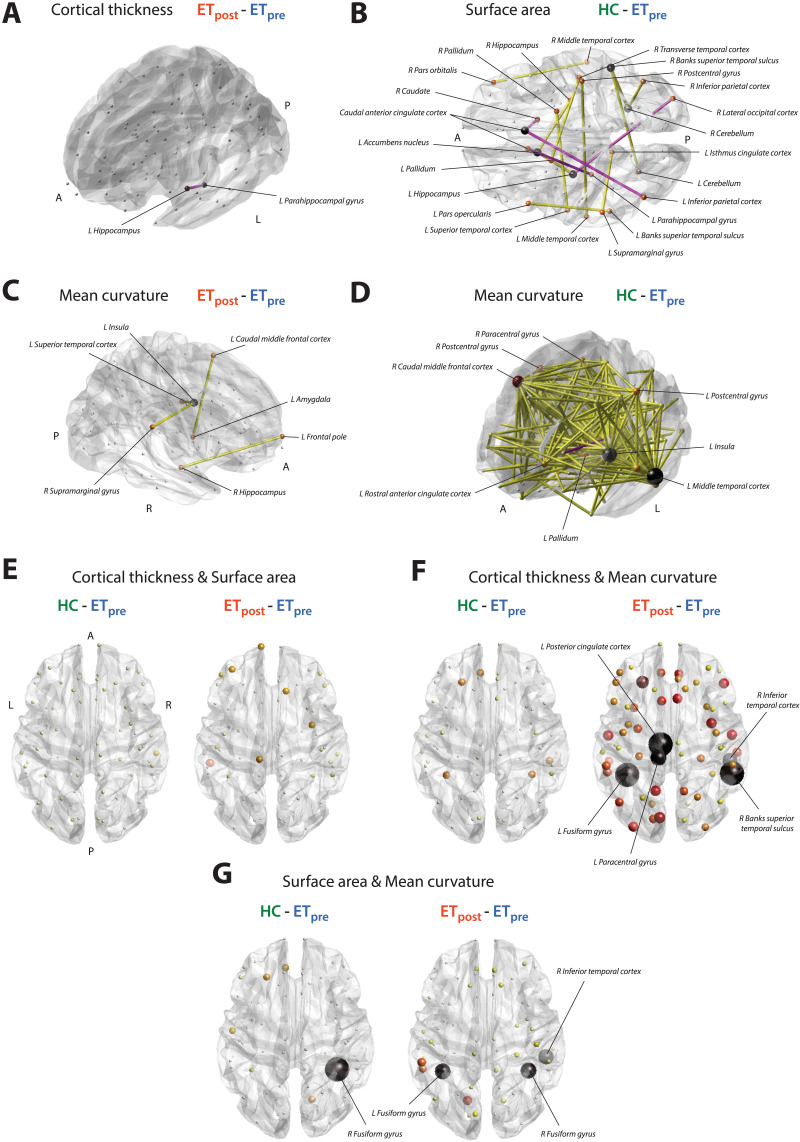
Graphical overview of significant findings. Significant structural covariance edges for cortical thickness and the ET_post_-ET_pre_ contrast (A), surface area and the HC-ET_pre_ contrast (B), and mean curvature and the ET_post_-ET_pre_ (C), and HC-ET_pre_ (D) contrasts. Positive-valued group differences are represented by yellow connections, and negative-values ones by pink connections. Nodal size and color are proportional to the number of emanating significant connections. For the cortical thickness/surface area (E), cortical thickness/mean curvature (F), and surface area/mean curvature (G) cross-measure analyses, overview of significance for the HC-ET_pre_ (left) and ET_post_-ET_pre_ (right) group differences. Nodal size and color are proportional to −log(*p* value), with black nodes the ones found significant in our analyses. P, posterior; R, right; A, anterior; L, left.

The one significant edge-wise SC difference between the ET_pre_ and ET_post_ groups involved the left parahippocampal gyrus and the left hippocampus (Figure 2A). Structural covariance was negligible at baseline (SC_pre_ = 0.25), and became negative valued following intervention (SC_post_ = −0.65, ΔSC = −0.91). This group difference was also related to the extent of tremor recovery (*β*_SC_ = 0.0069, μ_SC_ = 0.024).

For SA (Figure 1B), no difference across groups transpired at the global level. Fifteen connections reached significance between the HC and ET_pre_ groups (as summarized in Supporting Information Table S2 and displayed in Figure 2B), seven of which were related to the extent of tremor (*β*_SC_, μ_SC_ > 0). SC between the left supramarginal and left isthmus cingulate cortices, between the right pars orbitalis and right middle temporal cortex, and between the right postcentral gyrus and left middle temporal cortex was strongly positive valued in the HC group but close to zero in patients with ET. SC between the right caudate and right caudal anterior cingulate cortex was largely negative in HCs, but close to zero in the ET_pre_ group. SC between the left parahippocampal gyrus and left caudal anterior cingulate cortex, and between the right caudal anterior cingulate and the left inferior parietal cortices was conversely only present (and positive valued) in patients with ET. Finally, SC between the left pars opercularis and the left Banks superior temporal sulcus showed opposite sign in the HC (positive) and ET_pre_ (negative) groups.

For MC (Figure 1C), SC was consistently greater in magnitude in HC subjects compared to patients with ET both before and after the intervention (mean SC of 0.41 ± 0.35, 0.3 ± 0.35, and 0.22 ± 0.24, respectively; ET_pre_-HC *z* = −14.15, *p* < 0.001; ET_post_-HC *z* = −28.5107, *p* < 0.001). In addition, the ET_post_ group also exhibited overall lower SC values compared to the ET_pre_ one (ET_post_-ET_pre_ *z* = −11.2123, *p* < 0.001).

This resulted in four connections reaching significance for the ET_post_-ET_pre_ contrast (Figure 2C), between the left insula and left superior temporal cortex (SC_pre_ = 0.19, SC_post_ = 0.77, ΔSC = 0.58, *p* = 0.023, *β*_SC_ = 0.0002, μ_SC_ = −0.0062), right supramarginal gyrus and left insula (SC_pre_ = 0.13, SC_post_ = 0.86, ΔSC = 0.73, *p* = 0.043, *β*_SC_ = −0.003, μ_SC_ = −0.0055), left amygdala and left caudal middle frontal cortex (SC_pre_ = −0.33, SC_post_ = 0.57, ΔSC = 0.9, *p* = 0.023, *β*_SC_ = 0.0032, μ_SC_ = −0.011), and right hippocampus and left frontal pole (SC_pre_ = −0.38, SC_post_ = 0.51, ΔSC = 0.89, *p* = 0.043, *β*_SC_ = −0.025, μ_SC_ = −0.12). In all cases, structural covariance became significant following the intervention, but there was no association with the extent of tremor recovery.

In the HC-ET_pre_ case, there was a broad pattern of 195 significant connections, summarized in Supporting Information Table S3 and Figure 2D. Fifty-three of them showed an association with the extent of tremor, with the most implicated regions including the left inferior temporal cortex (showcased five times), left middle temporal cortex (17) and right caudal middle frontal cortex (11).

### Cross-Modality Analysis

The investigation of cross-modality relationships revealed, for the interactions between CT and SA, respective average covariance values across regions of −0.37 ± 0.25, −0.3 ± 0.2, and −0.4 ± 0.2 in the HC, ET_pre_, and ET_post_ groups. The ET_post_-ET_pre_ group difference was significant (Wilcoxon rank sum test’s *z* = −2.81, *p* = 0.045), but no individual regional interaction survived multiple testing correction.

**Figure 3.**
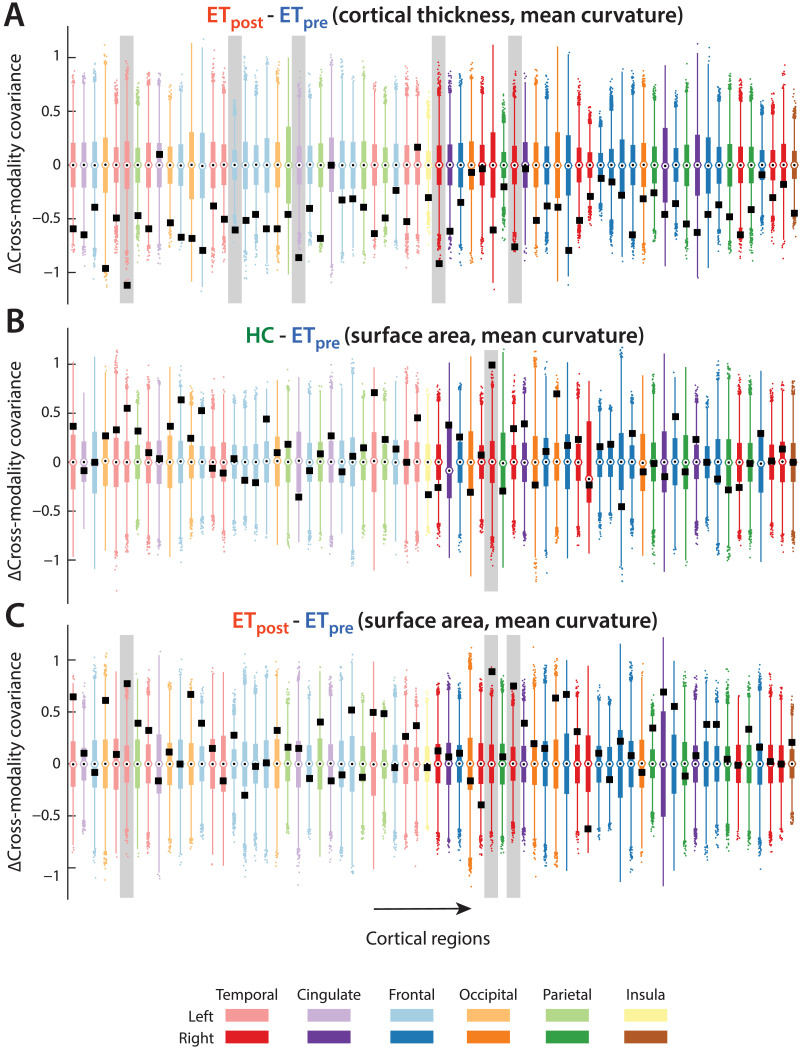
Cross-property covariance relationships. For the relationship between cortical thickness and mean curvature for the ET_post_-ET_pre_ contrast (A), and for the relationship between surface area and mean curvature for the HC-ET_pre_ (B), and ET_post_-ET_pre_ (C) group differences, cross-property covariance differences between groups (black squares) and associated null distributions (box plots) for all 68 cortical regions from the Desikan-Killiany atlas. Color coding of the box plots denotes brain lobes. Significant cases are highlighted by a light gray box in the background.

For the interplay between CT and MC, covariance was on the whole negligible in the HC (−0.1 ± 0.27) and ET_pre_ (0.03 ± 0.26) groups, but became negative valued following the intervention (−0.42 ± 0.2). This was confirmed by significant ET_post_-HC (*z* = −6.59, *p* < 0.001) and ET_post_-ET_pre_ (*z* = −8.14, *p* < 0.001) group differences. Five regions reached significance for the ET_post_-ET_pre_ contrast (Figure 3A and Figure 2F): the left fusiform gyrus (*C*_pre_ = 0.44, *C*_post_ = −0.68, Δ*C* = −1.12, *p* = 0), the left paracentral gyrus (*C*_pre_ = −0.075, *C*_post_ = −0.68, Δ*C* = −0.61, *p* = 0.0034), the left posterior cingulate cortex (*C*_pre_ = 0.17, *C*_post_ = −0.69, Δ*C* = −0.86, *p* = 0), the right Banks superior temporal sulcus (*C*_pre_ = 0.24, *C*_post_ = −0.67, Δ*C* = −0.91, *p* = 0), and the right inferior temporal cortex (*C*_pre_ = 0.11, *C*_post_ = −0.65, Δ*C* = −0.76, *p* = 0.0054). In all cases, the cross-modality covariance expectedly became strongly negative valued following the intervention.

When probing the relationships between SA and MC, cross-measure covariance was mildly positive in the HC group (0.18 ± 0.22), close to zero in the ET_pre_ group (0.06 ± 0.24), and increased after the intervention (0.25 ± 0.21). Accordingly, significant group differences were found in the HC-ET_pre_ (*z* = 3.04, *p* = 0.022) and ET_post_-ET_pre_ (*z* = 4.49, *p* < 0.001) cases (Figure 2G). At the level of individual regions, for the HC-ET_pre_ contrast (Figure 3B), the right fusiform gyrus showed a positive covariance between modalities in HC subjects, but only a mild negative one in patients with ET (*C*_HC_ = 0.66, *C*_pre_ = −0.34, Δ*C* = 0.99, *p* = 0). For the ET_post_-ET_pre_ contrast (Figure 3C), there was a transition toward positive-valued covariance upon intervention in the left fusiform gyrus (*C*_pre_ = −0.33, *C*_post_ = 0.44, Δ*C* = 0.77, *p* = 0.02), the right fusiform gyrus (*C*_pre_ = −0.34, *C*_post_ = 0.55, Δ*C* = 0.88, *p* = 0.02), and the right inferior temporal cortex (*C*_pre_ = −0.18, *C*_post_ = 0.57, Δ*C* = 0.75, *p* = 0.027).

## DISCUSSION

### Motor and Nonmotor Characteristics of ET Are Captured by SCA

We examined structural covariance patterns in patients with ET before and after thalamotomy, as well as preintervention ET cases compared to age-matched HCs. In doing so, we considered three popular and complementary morphometric properties (Harnett et al., 2020; Lerch et al., 2006; Raznahan et al., 2011; Sanabria-Diaz et al., 2010; Yang et al., 2013; Yang et al., 2016): CT, SA, and MC.

At the whole-brain level, we observed very different structural covariance patterns across them: for CT, SC was overall lower in ET_pre_ subjects compared to HCs. It then reverted following the intervention, denoting large-scale recovery. This did, however, not translate into significant individual SC edges (likely owing to the multiple testing approach and too small changes to be captured with our statistical power), as only the structural covariance between the left hippocampus and left parahippocampal cortex was found to differ between the ET_pre_ and ET_post_ groups, in a way that was also associated with the extent of tremor recovery. The appearance of a negative-valued SC, while it was strongly positive in the HC group (SC_HC_ = 0.5406), hints at an alternative mechanism of tremor recovery in which the geometry of both structures becomes jointly regulated (i.e., if one is thicker/larger in a subject, then the other tends to be thinner/smaller).

For SA, there was no clear difference in whole-brain SC patterns across groups. However, spatially subtle differences could be captured upon edge-wise analysis between the HC and ET_pre_ groups. In some cases, structural covariance disappeared in patients with ET, while in others it was only seen then. The supramarginal and postcentral cortices, but also the caudal anterior cingulate cortex, at the interface between frontal executive and parietal sensorimotor systems (Taylor et al., 2007), and the caudate, canonically linked to movement planning (Villablanca, 2010), were all involved areas, highlighting that ET induces complex structural covariance rearrangements within a broad network of motion-related brain areas.

The pattern observed for MC was particularly intriguing, as SC was lower in ET_pre_ subjects compared to HCs, and then continued to globally decrease following the intervention. Thus, the observed MC changes could relate to aspects of ET that are not purely motor, such as associated cognitive deficits and concomitant depression. Lending support to this theory, at the level of the ET_post_-ET_pre_ group differences, the four unraveled connections did not show an association to the extent of motor recovery, and involved frontal brain centers, implicated in executive functions (Braun et al., 2015; Stuss, 2011), as well as the amygdala and the insula, respectively most well-known for their role in emotional processes (Phelps, 2006) and salience monitoring (Menon & Uddin, 2010). In previous studies, ET patients with depression showed larger superior frontal nodal efficiency (Li et al., 2021), and regional homogeneity was increased in the middle prefrontal cortex (Duan et al., 2021), demonstrating that frontal alterations should be expected in ET patients with depression. While it could thus be that our results relate to nonmotor characteristics of ET, the lack of dedicated ratings in our data precludes further investigation at this stage.

To summarize, our edge-wise analysis enabled us to pinpoint a set of structural covariance relationships altered upon the presence of ET, or that have been modified following Vim thalamotomy, across a set of three morphometric properties. Many of the implicated areas relate to movement planning or execution, as could be expected in the context of ET; in these cases, we also observed an association with the extent of motor symptoms (for the HC-ET_pre_ comparison) or motor recovery (for the ET_post_-ET_pre_ one). Furthermore, some of the unraveled connections also involved regions modulating nonmotor functions, such as emotional control or attention. As no association to motor symptoms was found in these cases, these features are likely indicative of nonmotor attributes of ET.

### Evidence for a Role of Visual Cortical Areas in ET

In addition to the above observations, our results also converged on a picture in which brain areas linked to visual functions could be related to the occurrence of ET, or to plasticity following Vim thalamotomy. Recall that for CT, the only significant edge (in the ET_post_-ET_pre_ case) linked the left parahippocampal gyrus and hippocampus; the parahippocampal gyrus has been implicated in episodic memory, but also in visuospatial processing (Aminoff et al., 2013), while the hippocampus relates to memory, spatial navigation, and cognition (Lisman et al., 2017). Fittingly, a previous VBM study of ours also related the left parahippocampal place area to TSTH improvement following intervention (Tuleasca et al., 2017).

For MC, between the HC and ET_pre_ groups, the most involved area in the 53 significant connections showing an association with tremor severity was the left middle temporal cortex, which includes the famous area MT that encodes the perception of movement within the visual field (Born & Bradley, 2005). The right caudal middle frontal cortex was the second most implicated region, and may relate to alterations at the level of the frontal eye fields, which control eye movements (Robinson & Fuchs, 1969) and contribute to visual selection (Muggleton et al., 2003).

Our second analysis, in which we considered the interplay across morphometric properties, further corroborates the importance of visual areas in ET. Only one feature contrasted HC and ET_pre_ subjects: the covariance between SA and MC in the right fusiform gyrus, an area involved in higher order visual functions (Weiner & Zilles, 2016). The strong positive-valued covariance across modalities seen in HCs disappeared in patients with ET, and was accompanied by broadly lower cross-property covariance in the diseased group.

Interestingly, this diffuse weakening renormalized following the intervention, and interactions between the other pairs of properties also changed: CT and SA regained stronger anticorrelation akin to that seen in HCs, and there was a marked appearance of negative-valued CT/MC covariance. Thus, in addition to the renormalization of CT/SA and SA/MC interactions, the development of a more antagonistic relationship between CT and MC appears to be an alternative mechanism of recovery.

At the regional level, the paracentral gyrus, the right inferior temporal cortex—important for visual perception (Miyashita, 1993)—and the bilateral fusiform gyrus were involved in these changes following stereotactic radiosurgical thalamotomy. Such findings are supported by a previous resting-state functional MRI work from our group, in which a component reminiscent of a salience network showed altered interconnectivity with the right fusiform gyrus and middle temporal visual area (Tuleasca, Regis, et al., 2018). Further highlighting the importance of visual regions in postinterventional tremor recovery, another recent study on Parkinson’s disease patients evidenced a correlation between tremor improvement following MRI-guided focused ultrasound thalamotomy and signal intensity in the left occipital cortex (Xiong et al., 2021).

Taken together, these results suggest that cross-regional or cross-property dependences involving brain regions mediating high-level visual functions are tied to ET. This extends a past morphometric report in which a direct relationship between posttherapeutic improvement in tremor of the treated hand and pretherapeutic GM density of the right visual association area was evidenced (Tuleasca, Witjas, et al., 2018). It also raises the question of potentially targeting visual networks in the near future.

### Limitations and Future Perspectives

It is important to acknowledge the limitations of our study: in particular, the number of subjects at our disposal remains quite low, likely precluding the unraveling of finer ET-related structural covariance patterns. However, it should be remembered that the patients analyzed therein belong to a subgroup of drug-resistant individuals only; in addition, morphometric data could be collected at two time points before and 1 year after thalamotomy. Of course, it would be interesting to monitor how recovery continues to evolve over the course of time, with an even more longitudinal design. One might hypothesize that additional structural covariance adjustments would then start developing, although empirically, clinical and radiological changes remain minimal.

Another potential limitation is the dependence of our results on the parcellation that was used for the analyses; indeed, a finer-grained atlas could perhaps provide yet more spatially accurate results. However, the number of statistical tests conducted in parallel would then also dramatically increase, and so would the extent of dependences between spatially neighboring areas. More advanced statistical correction methods would then have to be deployed for appropriate analysis; see, for example, Meskaldji et al. (2015).

In addition, care should be taken in the mechanistic interpretation of structural covariance findings: positive-valued edges are only sometimes paralleled by physical wiring (perhaps due to the impact of mutually trophic effects), while factors such as the (anti)coherence of neural activity (leading to similar or opposite plastic changes depending on the sign of the interaction) may be at play in other cases (Gong et al., 2012).

Covariance between different morphometric properties should also be contemplated with caution; indeed, the interactions across measures are largely reshaped throughout the lifespan. For example, while CT and SA evolve with different timings and characteristic regional patterns (Raznahan et al., 2011; Wierenga et al., 2014), they do so in a tightly intertwined and nontrivial manner (Schnack et al., 2015). While our morphometric estimates were regressed out for age and only spanned a relatively narrow age range, further nonlinear effects could still have remained present in the data.

Our cross-measure results on HCs were in line with previous research: cortical gyrification has been positively/negatively associated with SA/CT, and a negative relationship between CT and SA has also been evidenced throughout the lifespan, including in older adults as assessed here (Hogstrom et al., 2013). Mechanistically speaking, a larger SA is believed to facilitate resource allocation, computational capacity, and functional specificity, while cortical thinning may be associated to pruning and the potentiation of information flow within cortical columns (Tadayon et al., 2020). We observed this antagonism to be restored to a level comparable to HCs in patients with ET following intervention, and the same held true for the positive relationship between SA and MC. The negative relationship between CT and MC was, however, strengthened much beyond its level in HCs. Cortical thinning and concomitant greater gyral complexity have been observed in anxiety disorder and 22q11.2 deletion syndrome (Bearden et al., 2009; Molent et al., 2018); in future work, it will be interesting to determine what are the neurobiological mechanisms at play, and whether this antagonistic coupling in ET may relate to subtle anxiety- or cognition-related deficits known to occur in the disease (Bermejo-Pareja, 2011).

Methodologically speaking, it will also be interesting to translate the proposed analytical approach to the network-level spatial scale: to achieve this, instead of focusing on individual cross-regional edges, graph theoretical metrics (Rubinov & Sporns, 2010) could be computed from whole-brain structural covariance patterns and compared between groups. Given the fact that ET is widely regarded as a network-level disorder (Raethjen & Deuschl, 2012), such an analysis can be expected to further illuminate our understanding of the disease, and of brain plasticity following intervention.

## ACKNOWLEDGMENTS

Dr. Tuleasca gratefully acknowledges the receipt of a Young Researcher in Clinical Research Grant (“Jeune Chercheur en Recherche Clinique”) from the University of Lausanne (UNIL), Faculty of Biology and Medicine (FBM), and the Lausanne University Hospital (CHUV). The authors also wish to thank Andrea Sullca for her help in compiling the presented results.

## SUPPORTING INFORMATION

Supporting information for this article is available at https://doi.org/10.1162/netn_a_00253.

## AUTHOR CONTRIBUTIONS

Thomas Bolton: Formal analysis; Investigation; Methodology; Software; Validation; Visualization; Writing – original draft; Writing – review & editing. Dimitri Van De Ville: Supervision; Writing – review & editing. Jean Régis: Conceptualization; Data curation; Funding acquisition; Supervision. Tatiana Witjas: Data curation; Project administration. Nadine Girard: Data curation; Project administration. Marc Levivier: Conceptualization; Data curation; Funding acquisition; Project administration; Resources; Supervision. Constantin Tuleasca: Conceptualization; Data curation; Funding acquisition; Investigation; Resources; Supervision; Validation; Writing – review & editing.

## FUNDING INFORMATION

Constantin Tuleasca, Université de Lausanne (https://dx.doi.org/10.13039/501100006390).

## TECHNICAL TERMS

Essential tremor (ET): A brain disorder whose clinical symptoms include tremor of the upper limbs and subtle cognitive deficits.
Tremor network: A set of interconnected brain areas believed to be involved in the onset and progression of ET.
Brain morphometry: The quantitative analysis of the shape and dimensions of brain structures, including local volume, thickness, surface area, and curvature.
Structural covariance analysis: An analytical approach in which the cross-regional covariance in a morphometric measure of interest is quantified across subjects.
Tremor Score on Treated Hand (TSTH): A clinical score that quantifies the extent of tremor in a patient, with higher values denoting greater motor impairment.
FreeSurfer: An open source brain imaging package enabling the extraction of morphometric estimates from structural neuroimaging data.
Nodal efficiency: A graph theoretical measure that quantifies how fast information can spread from a given node to all others in the network.
Regional homogeneity: A metric that quantifies the temporal coherence between a set of a given voxel’s spatial neighbors.
Stereotactic radiosurgical thalamotomy: A surgical intervention in which the thalamus is locally lesioned with ionizing radiation to alleviate tremor in drug-resistant patients.
