# Supplementary material for: Morphometric features of drug-resistant essential tremor and recovery after stereotactic radiosurgical thalamotomy
Source: Netw Neurosci. 2022 Jul 1;6(3):850–69. doi: 10.1162/netn_a_00253 (PMC9810368; doi:10.1162/netn_a_00253)
Supplement: Supplementary file 1 [file netn-06-850-s001.pdf]

Bolton, T., Van De Ville, D., Régis, J., Witjas, T., Girard, N., Levivier, M., Tuleasca, C. (2022). Supporting information for “Morphometric features of drug-resistant essential tremor and recovery after stereotactic radiosurgical thalamotomy.” *Network Neuroscience*, 6(3): 850–869. https://doi.org/netn_a_00253

Supplementary Table 1: Regions of the considered atlas. All the regions analyzed in this work are listed alongside their index and the hemisphere to which they belong.

Index	Hemisphere	Name
1	Left	Banks superior temporal sulcus
2	Left	Caudal anterior cingulate cortex
3	Left	Caudal middle frontal cortex
4	Left	Cuneus
5	Left	Entorhinal cortex
6	Left	Fusiform gyrus
7	Left	Inferior parietal cortex
8	Left	Inferior temporal cortex
9	Left	Isthmus cingulate cortex
10	Left	Lateral occipital cortex
11	Left	Lateral orbitofrontal cortex
12	Left	Lingual cortex
13	Left	Medial orbitofrontal cortex
14	Left	Middle temporal cortex
15	Left	Parahippocampal gyrus
16	Left	Paracentral gyrus
17	Left	Pars opercularis
18	Left	Pars orbitalis
19	Left	Pars triangularis
20	Left	Pericalcarine gyrus
21	Left	Postcentral gyrus
22	Left	Posterior cingulate cortex
23	Left	Precentral gyrus
24	Left	Precuneus
25	Left	Rostral anterior cingulate cortex
26	Left	Rostral middle frontal cortex
27	Left	Superior frontal cortex
28	Left	Superior parietal cortex
29	Left	Superior temporal cortex
30	Left	Supramarginal gyrus
31	Left	Frontal pole
32	Left	Temporal pole
33	Left	Trasverse temporal cortex
34	Left	Insula
35	Right	Banks superior temporal sulcus
36	Right	Caudal anterior cingulate cortex
37	Right	Caudal middle frontal cortex
38	Right	Cuneus
39	Right	Entorhinal cortex
40	Right	Fusiform gyrus
41	Right	Inferior parietal cortex
42	Right	Inferior temporal cortex
43	Right	Isthmus cingulate cortex

Bolton, T., Van De Ville, D., Régis, J., Witjas, T., Girard, N., Levivier, M., Tuleasca, C. (2022). Supporting information for "Morphometric features of drug-resistant essential tremor and recovery after stereotactic radiosurgical thalamotomy." *Network Neuroscience*. Advance publication. https://doi.org/netn_a_00253

44	Right	Lateral occipital cortex
45	Right	Lateral orbitofrontal cortex
46	Right	Lingual cortex
47	Right	Medial orbitofrontal cortex
48	Right	Middle temporal cortex
49	Right	Parahippocampal gyrus
50	Right	Paracentral gyrus
51	Right	Pars opercularis
52	Right	Pars orbitalis
53	Right	Pars triangularis
54	Right	Pericalcarine gyrus
55	Right	Postcentral gyrus
56	Right	Posterior cingulate cortex
57	Right	Precentral gyrus
58	Right	Precuneus
59	Right	Rostral anterior cingulate cortex
60	Right	Rostral middle frontal cortex
61	Right	Superior frontal cortex
62	Right	Superior parietal cortex
63	Right	Superior temporal cortex
64	Right	Supramarginal gyrus
65	Right	Frontal pole
66	Right	Temporal pole
67	Right	Trasverse temporal cortex
68	Right	Insula
69	Left	Cerebellar white matter
70	Left	Cerebellum
71	Left	Thalamus
72	Left	Caudate
73	Left	Putamen
74	Left	Pallidum
75	Left	Hippocampus
76	Left	Amygdala
77	Left	Accumbens nucleus
78	Right	Cerebellar white matter
79	Right	Cerebellum
80	Right	Thalamus
81	Right	Caudate
82	Right	Putamen
83	Right	Pallidum
84	Right	Hippocampus
85	Right	Amygdala
86	Right	Accumbens nucleus
87	-	Brainstem

Bolton, T., Van De Ville, D., Régis, J., Witjas, T., Girard, N., Levivier, M., Tuleasca, C. (2022). Supporting information for "Morphometric features of drug-resistant essential tremor and recovery after stereotactic radiosurgical thalamotomy." *Network Neuroscience*. Advance publication. https://doi.org/netn_a_00253

Supplementary Table 2: Significant connections for the HC - ET_{pre} contrast (surface area). The two implicated regions are reported alongside the difference statistic (ΔSC), the individual structural covariance values of each group (SC_{HC} and SC_{pre}), FDR-corrected *p*-values and the summarizing metrics β_{SC} and μ_{SC}. Connections with an association to the extent of tremor are highlighted in italics,

**HC- ET_{pre}**							
**Region 1**	**Region 2**	**ΔSC**	**SC_{HC}**	**SC_{pre}**	***p*-value**	**β_{SC}**	**μ_{SC}**
L Parahippocampal gyrus	*L Caudal anterior cingulate cortex*	*-0.7439*	*-0.2555*	*0.4885*	*0.0142*	*0.0089*	*0.0810*
L Pars opercularis	*L Banks superior temporal sulcus*	*0.9921*	*0.4589*	*-0.5332*	*0.0142*	*0.0064*	*0.0427*
L Supramarginal	*L Isthmus cingulate*	*0.7150*	*0.6808*	*-0.0343*	*0.0264*	*0.0168*	*0.0976*
R Caudal anterior cingulate	*L Inferior parietal cortex*	*-0.6341*	*-0.0697*	*0.5645*	*0.0264*	*0.0135*	*0.0639*
R Pars orbitalis	*R Middle temporal cortex*	*0.6878*	*0.5125*	*-0.1752*	*0.0266*	*0.0131*	*0.0534*
R Postcentral	*L Middle temporal cortex*	*0.7738*	*0.7196*	*-0.0542*	*0.0250*	*0.0157*	*0.0853*
L Cerebellum	R Banks superior temporal sulcus	0.8720	0.5674	-0.3046	0.0266	-0.0020	-0.0142
L Pallidum	R Transverse temporal cortex	0.9176	0.4822	-0.4354	0.0374	-0.0128	-0.0691
L Hippocampus	R Lateral occipital cortex	-0.9068	-0.5600	0.3468	0.0374	-0.0038	-0.0825
L Accumbens nucleus	L Hippocampus	0.9837	0.5402	-0.4435	0.0200	-0.0062	-0.0403
R Cerebellum	R Banks superior temporal sulcus	0.9305	0.5218	-0.4088	0.0142	-0.0004	-0.0079
R Cerebellum	R Inferior parietal cortex	0.8967	0.5453	-0.3513	0.0142	0.0009	-0.0187
R Caudate	*R Caudal anterior cingulate cortex*	*-1.1204*	*-0.8188*	*0.3016*	*0.0250*	*0.0201*	*0.0463*
**R Pallidum**	**L Superior temporal cortex**	**1.0318**	**0.3791**	**-0.6527**	**0.0050**	**-0.0005**	**-0.0193**
R Hippocampus	L Accumbens nucleus	1.1681	0.7407	-0.4275	0.0142	-0.0081	-0.0538

and edges significant with $p<0.01$ are displayed in bold. L: Left; R: Right.

Bolton, T., Van De Ville, D., Régis, J., Witjas, T., Girard, N., Levivier, M., Tuleasca, C. (2022). Supporting information for “Morphometric features of drug-resistant essential tremor and recovery after stereotactic radiosurgical thalamotomy.” *Network Neuroscience*. Advance publication. https://doi.org/netn_a_00253

Supplementary Table 3: Significant connections for the HC - ET_{pre} contrast (mean curvature).

The two implicated regions are reported alongside the difference statistic (ΔSC), the individual structural covariance values of each group (SC_{HC} and SC_{pre}), FDR-corrected *p*-values and the summarizing metrics β_{SC} and μ_{SC}. Connections with an association to the extent of tremor are highlighted in italics, and edges significant with $p<0.01$ are displayed in bold. L: Left; R: Right.

**HC - ET_{pre}**							
**Region 1**	**Region 2**	**ΔSC**	**SC_{HC}**	**SC_{pre}**	***p*-value**	**β_{SC}**	**μ_{SC}**
**L Entorhinal cortex**	**L Caudal anterior cingulate cortex**	**0.7266**	**0.6698**	**-0.0568**	**0.0038**	**-0.0111**	**-0.0097**
L Middle temporal cortex	L Banks suoperior temporal sulcus	0.5638	0.7235	0.1597	0.0215	-0.0078	-0.0370
L Middle temporal cortex	***L Caudal middle frontal cortex***	***0.5409***	***0.8863***	***0.3454***	***0.0055***	***0.0037***	***0.0215***
L Middle temporal cortex	*L Fusiform gyrus*	*0.5890*	*0.7643*	*0.1753*	*0.0144*	*0.0038*	*0.0218*
**L Middle temporal cortex**	**L Isthmus cingulate**	**0.6927**	**0.7726**	**0.0799**	**0.0061**	**-0.0047**	**0.0192**
L Middle temporal cortex	*L Lateral occipital cortex*	*0.6129*	*0.8369*	*0.2240*	*0.0277*	*0.0009*	*0.0275*
**L Middle temporal cortex**	**L Lateral orbitofrontal cortex**	**0.6120**	**0.8075**	**0.1955**	**0.0063**	**-0.0107**	**-0.0031**
**L Middle temporal cortex**	**L Lingual cortex**	**0.5982**	**0.7557**	**0.1575**	**0.0024**	**-0.0007**	**0.0228**
L Parahippocampal gyrus	*L Middle temporal cortex*	*0.7627*	*0.6870*	*-0.0757*	*0.0145*	*0.0013*	*0.0048*
L Paracentral gyrus	L Middle temporal cortex	0.4127	0.7641	0.3514	0.0277	-0.0050	-0.0073
L Pericalcarine gyrus	L Cuneus	-0.2442	0.7272	0.9715	0.0352	0.0004	-0.0006
L Postcentral gyrus	*L Inferior temporal cortex*	*0.4252*	*0.8407*	*0.4156*	*0.0223*	*0.0085*	*0.0448*
L Postcentral gyrus	***L Middle temporal cortex***	***0.5256***	***0.8681***	***0.3425***	***0***	***0.0023***	***0.0241***
L Postcentral gyrus	L Paracentral gyrus	0.1682	0.9392	0.7710	0.0206	-0.0039	-0.0170
L Posterior cingulate cortex	*L Caudal anterior cingulate cortex*	*0.7844*	*0.6857*	*-0.0987*	*0.0208*	*0.0023*	*0.0191*
L Precentral gyrus	L Inferior temporal cortex	0.4179	0.8168	0.3989	0.0211	-0.0018	0.0222
**L Precentral gyrus**	**L Middle temporal cortex**	**0.5172**	**0.8417**	**0.3245**	**0.0011**	**-0.0005**	**0.0151**
L Rostral anterior cingulate cortex	L Isthmus cingulate	0.5935	0.6266	0.0331	0.0283	-0.0025	0.0439
L Rostral anterior cingulate cortex	*L Middle temporal cortex*	*0.5404*	*0.7207*	*0.1802*	*0.0223*	*0.0042*	*0.0360*
L Rostral anterior cingulate cortex	L Paracentral gyrus	0.4819	0.6612	0.1793	0.0361	-0.0052	-0.0054
**L Rostral anterior cingulate cortex**	**L Postcentral gyrus**	**0.6068**	**0.7813**	**0.1746**	**0.0029**	**-0.0121**	**-0.0111**
L Rostral anterior cingulate cortex	L Precentral gyrus	0.5031	0.7501	0.2470	0.0300	-0.0072	0.0015
L Rostral middle frontal cortex	*L Pericalcarine gyrus*	*-0.4451*	*0.3050*	*0.7500*	*0.0283*	*0.0030*	*0.0102*
L Superior parietal cortex	L Inferior temporal cortex	0.5860	0.8023	0.2163	0.0418	-0.0002	0.0155
L Superior parietal cortex	L Middle temporal cortex	0.6183	0.8399	0.2216	0.0136	-0.0048	-0.0140
L Superior parietal cortex	L Postcentral gyrus	0.1567	0.9496	0.7929	0.0403	-0.0067	-0.0210
L Superior temporal cortex	L Inferior temporal cortex	0.3388	0.8497	0.5109	0.0188	-0.0066	-0.0118
**L Superior temporal cortex**	**L Middle temporal cortex**	**0.5179**	**0.9021**	**0.3843**	**0.0011**	**-0.0037**	**0.0104**
L Superior temporal cortex	L Postcentral gyrus	0.1842	0.9005	0.7162	0.0349	-0.0064	-0.0145
L Superior temporal cortex	***L Rostral anterior cingulate cortex***	***0.7402***	***0.7846***	***0.0443***	***0.0020***	***0.0047***	***0.0432***
L Superior temporal cortex	L Superior parietal cortex	0.3552	0.8700	0.5148	0.0185	-0.0033	0.0012
L Supramarginal gyrus	L Fusiform gyrus	0.3879	0.8472	0.4594	0.0356	-0.0053	-0.0110
L Supramarginal gyrus	*L Middle temporal cortex*	*0.5027*	*0.7141*	*0.2114*	*0.0125*	*0.0060*	*0.0229*
L Temporal pole	L Supramarginal gyrus	0.6415	0.5804	-0.0611	0.0263	-0.0104	-0.0295
L Transverse temporal cortex	***L Middle temporal cortex***	***0.5252***	***0.7912***	***0.2660***	***0.0071***	***0.0022***	***0.0085***
L Insula	L Caudal middle frontal cortex	0.6114	0.8097	0.1984	0.0215	-0.0028	0.0345
**L Insula**	**L Fusiform gyrus**	**0.6234**	**0.7364**	**0.1130**	**0.0016**	**-0.0055**	**-0.0343**

Bolton, T., Van De Ville, D., Régis, J., Witjas, T., Girard, N., Levivier, M., Tuleasca, C. (2022). Supporting information for "Morphometric features of drug-resistant essential tremor and recovery after stereotactic radiosurgical thalamotomy." *Network Neuroscience*. Advance publication. https://doi.org/netn_a_00253

L Insula	L Inferior parietal cortex	0.5992	0.6802	0.0809	0.0206	-0.0122	-0.0176
L Insula	***L Inferior temporal cortex***	***0.7925***	***0.7223***	***-0.0702***	***0.0081***	***0.0008***	***0.0305***
L Insula	L Isthmus cingulate	0.6940	0.7010	0.0071	0.0125	-0.0194	-0.0317
**L Insula**	**L Lateral orbitofrontal cortex**	**0.7228**	**0.8068**	**0.0840**	**0.0094**	**-0.0094**	**-0.0313**
L Insula	*L Middle temporal cortex*	*0.6140*	*0.7428*	*0.1287*	*0.0206*	*0.0009*	*0.0111*
**L Insula**	**L Paracentral gyrus**	**0.6691**	**0.7665**	**0.0974**	**0.0087**	**-0.0086**	**-0.0233**
L Insula	L Postcentral gyrus	0.5110	0.7500	0.2389	0.0356	-0.0144	-0.0354
**L Insula**	**L Precentral gyrus**	**0.6331**	**0.8243**	**0.1913**	**0.0040**	**-0.0079**	**-0.0038**
L Insula	L Superior parietal cortex	0.4948	0.7286	0.2339	0.0411	-0.0075	-0.0092
L Insula	L Superior temporal cortex	0.5346	0.7263	0.1917	0.0424	-0.0039	0.0125
L Insula	L Temporal pole	0.7639	0.6649	-0.0990	0.0435	-0.0018	0.0061
R Banks superior temporal sulcus	*L Lateral occipital cortex*	*0.5049*	*0.8020*	*0.2971*	*0.0463*	*0.0015*	*0.0499*
R Banks superior temporal sulcus	L Postcentral gyrus	0.4153	0.7300	0.3147	0.0386	-0.0003	0.0158
R Banks superior temporal sulcus	*L Superior temporal cortex*	*0.4569*	*0.6973*	*0.2404*	*0.0481*	*0.0060*	*0.0553*
R Caudal middle frontal cortex	*L Caudal middle frontal cortex*	*0.4168*	*0.9181*	*0.5013*	*0.0159*	*0.0138*	*0.0351*
R Caudal middle frontal cortex	L Fusiform gyrus	0.5716	0.7174	0.1458	0.0300	-0.0047	-0.0200
R Caudal middle frontal cortex	***L Isthmus cingulate***	***0.6010***	***0.7600***	***0.1590***	***0.0011***	***0.0135***	***0.0230***
**R Caudal middle frontal cortex**	**L Lateral orbitofrontal cortex**	**0.5139**	**0.9151**	**0.4012**	**0.0073**	**-0.0042**	**-0.0511**
R Caudal middle frontal cortex	L Parahippocampal gyrus	0.5473	0.6080	0.0607	0.0451	-0.0005	-0.0038
R Caudal middle frontal cortex	L Paracentral gyrus	0.3421	0.8032	0.4612	0.0409	-0.0024	-0.0167
R Caudal middle frontal cortex	L Pars opercularis	0.4597	0.8039	0.3442	0.0463	-0.0116	-0.0298
R Caudal middle frontal cortex	*L Postcentral gyrus*	*0.3490*	*0.8864*	*0.5374*	*0.0106*	*0.0144*	*0.0351*
**R Caudal middle frontal cortex**	**L Precentral gyrus**	**0.4578**	**0.9176**	**0.4597**	**0.0024**	**0.0031**	**-0.0057**
R Caudal middle frontal cortex	L Superior parietal cortex	0.4137	0.8505	0.4368	0.0195	-0.0037	-0.0181
**R Caudal middle frontal cortex**	**L Superior temporal cortex**	**0.5061**	**0.8433**	**0.3373**	**0.0067**	**0.0006**	**-0.0079**
**R Caudal middle frontal cortex**	**L Insula**	**0.7529**	**0.8162**	**0.0633**	**0.0038**	**-0.0095**	**-0.0221**
R Cuneus	*L Middle temporal cortex*	*0.6266*	*0.6857*	*0.0591*	*0.0289*	*0.0033*	*0.0345*
**R Entorhinal cortex**	**L Middle temporal cortex**	**0.7587**	**0.7241**	**-0.0346**	**0.0071**	**-0.0002**	**0.0401**
R Entorhinal cortex	*R Caudal middle frontal cortex*	*0.5283*	*0.7517*	*0.2234*	*0.0109*	*0.0050*	*0.0102*
R Fusiform gyrus	*L Middle temporal cortex*	*0.3752*	*0.8082*	*0.4330*	*0.0451*	*0.0010*	*0.0113*
**R Fusiform gyrus**	**L Insula**	**0.7951**	**0.7671**	**-0.0280**	**0.0020**	**-0.0137**	**-0.0086**
R Inferior parietal cortex	*L Inferior temporal cortex*	*0.6181*	*0.8055*	*0.1874*	*0.0356*	*0.0013*	*0.0224*
R Inferior parietal cortex	***L Middle temporal cortex***	***0.6738***	***0.8374***	***0.1636***	***0.0065***	***0.0025***	***0.0136***
**R Inferior parietal cortex**	**L Insula**	**0.5845**	**0.7662**	**0.1817**	**0.0058**	**-0.0009**	**0.0249**
R Inferior parietal cortex	*R Caudal middle frontal cortex*	*0.5516*	*0.8410*	*0.2894*	*0.0157*	*0.0014*	*0.0014*
**R Inferior temporal cortex**	**L Insula**	**0.6706**	**0.7219**	**0.0513**	**0.0055**	**-0.0054**	**-0.0167**
R Inferior temporal cortex	***R Caudal middle frontal cortex***	***0.4154***	***0.8795***	***0.4641***	***0.0058***	***0.0175***	***0.0618***
R Inferior temporal cortex	R Entorhinal gyrus	0.4839	0.7424	0.2584	0.0327	-0.0017	0.0209
**R Isthmus cingulate**	**L Middle temporal cortex**	**0.5872**	**0.7945**	**0.2073**	**0.0075**	**-0.0030**	**0.0081**
R Isthmus cingulate	L Insula	0.6748	0.7882	0.1133	0.0134	-0.0077	0.0179
R Isthmus cingulate	*R Caudal middle frontal cortex*	*0.5556*	*0.8114*	*0.2558*	*0.0215*	*0.0081*	*0.0136*
**R Lateral occipital cortex**	**L Middle temporal cortex**	**0.6011**	**0.8977**	**0.2967**	**0.0083**	**-0.0049**	**-0.0048**
R Lateral occipital cortex	L Insula	0.5948	0.6467	0.0519	0.0424	-0.0025	0.0516
R Lateral orbitofrontal cortex	L Middle temporal cortex	0.5198	0.7216	0.2018	0.0451	-0.0015	0.0310
**R Lateral orbitofrontal cortex**	**R Caudal middle frontal cortex**	**0.5037**	**0.8514**	**0.3477**	**0.0083**	**0.0016**	**-0.0177**
R Lingual cortex	L Middle temporal cortex	0.4350	0.7538	0.3188	0.0481	-0.0006	0.0270
R Lingual cortex	L Parahippocampal gyrus	0.5761	0.7919	0.2158	0.0364	0.0010	-0.0312
R Lingual cortex	L Insula	0.6681	0.7226	0.0544	0.0127	-0.0054	0.0232

Bolton, T., Van De Ville, D., Régis, J., Witjas, T., Girard, N., Levivier, M., Tuleasca, C. (2022). Supporting information for “Morphometric features of drug-resistant essential tremor and recovery after stereotactic radiosurgical thalamotomy.” *Network Neuroscience*. Advance publication. https://doi.org/netn_a_00253

R Middle temporal cortex	L Middle temporal cortex	0.4769	0.8946	0.4176	0.0127	-0.0111	-0.0465
R Middle temporal cortex	L Postcentral gyrus	0.3390	0.8538	0.5148	0.0423	-0.0010	0.0020
R Middle temporal cortex	L Rostral anterior cingulate cortex	0.4324	0.7579	0.3255	0.0223	-0.0175	-0.0526
R Middle temporal cortex	L Superior temporal cortex	0.3824	0.8178	0.4354	0.0195	-0.0049	-0.0065
R Middle temporal cortex	*L Supramarginal gyrus*	*0.4273*	*0.7870*	*0.3596*	*0.0391*	*0.0063*	*0.0253*
R Middle temporal cortex	L Insula	0.6594	0.6705	0.0112	0.0150	-0.0058	0.0267
R Middle temporal cortex	R Caudal middle frontal cortex	0.3986	0.8453	0.4467	0.0138	-0.0032	-0.0418
R Parahippocampal gyrus	L Caudal anterior cingulate cortex	0.5265	0.4721	-0.0544	0.0397	-0.0039	-0.0290
R Parahippocampal gyrus	***L Middle temporal cortex***	***0.7251***	***0.7267***	***0.0016***	***0.0020***	***0.0031***	***0.0340***
R Parahippocampal gyrus	L Insula	0.5106	0.6848	0.1742	0.0260	-0.0042	-0.0138
R Parahippocampal gyrus	*R Caudal middle frontal cortex*	*0.6052*	*0.6962*	*0.0910*	*0.0159*	*0.0056*	*0.0144*
R Paracentral gyrus	*L Banks superior temporal sulcus*	*0.5536*	*0.7136*	*0.1600*	*0.0283*	*0.0054*	*0.0209*
R Paracentral gyrus	***L Inferior temporal cortex***	***0.6591***	***0.7467***	***0.0875***	***0.0073***	***0.0010***	***0.0347***
**R Paracentral gyrus**	**L Middle temporal cortex**	**0.6167**	**0.7498**	**0.1331**	**0.0040**	**-0.0003**	**0.0138**
**R Paracentral gyrus**	**L Paracentral gyrus**	**0.2146**	**0.9659**	**0.7513**	**0.0011**	**0.0004**	**-0.0051**
R Paracentral gyrus	L Rostral anterior cingulate cortex	0.3680	0.6408	0.2728	0.0356	-0.0044	-0.0030
R Paracentral gyrus	L Superior temporal cortex	0.3085	0.8212	0.5127	0.0206	-0.0004	0.0142
R Paracentral gyrus	L Insula	0.5208	0.7207	0.1999	0.0219	-0.0097	-0.0097
R Paracentral gyrus	***R Caudal middle frontal cortex***	***0.4766***	***0.7889***	***0.3123***	***0.0095***	***0.0049***	***0.0123***
R Paracentral gyrus	*R Lingual cortex*	*0.3183*	*0.8859*	*0.5676*	*0.0208*	*0.0034*	*0.0240*
R Pars opercularis	L Pars opercularis	0.4046	0.8572	0.4527	0.0260	-0.0178	-0.0376
R Pars orbitalis	L Insula	0.7176	0.6976	-0.0200	0.0439	-0.0101	-0.0106
R Pars triangularis	L Inferior temporal cortex	0.4451	0.7472	0.3021	0.0195	-0.0034	-0.0097
R Pars triangularis	L Middle temporal cortex	0.4307	0.7352	0.3045	0.0403	0.0010	-0.0111
R Pars triangularis	L Pars opercularis	0.4421	0.8240	0.3819	0.0376	-0.0146	-0.0279
**R Pars triangularis**	**L Temporal pole**	**0.5679**	**0.7675**	**0.1995**	**0.0094**	**-0.0011**	**-0.0031**
R Pars triangularis	R Middle temporal cortex	0.3190	0.7491	0.4300	0.0463	-0.0005	-0.0067
R Pericalcarine gyrus	L Insula	0.6234	0.7062	0.0828	0.0127	-0.0004	0.0559
R Postcentral gyrus	L Inferior temporal cortex	0.3562	0.7491	0.3929	0.0285	-0.0193	-0.0399
**R Postcentral gyrus**	**L Middle temporal cortex**	**0.4736**	**0.7761**	**0.3025**	**0**	**-0.0133**	**-0.0299**
R Postcentral gyrus	L Parahippocampal gyrus	0.5470	0.6639	0.1168	0.0110	-0.0014	-0.0444
R Postcentral gyrus	L Paracentral gyrus	0.2967	0.8906	0.5939	0.0144	-0.0130	-0.0434
**R Postcentral gyrus**	**L Postcentral gyrus**	**0.2564**	**0.8938**	**0.6374**	**0.0011**	**-0.0249**	**-0.0713**
**R Postcentral gyrus**	**L Precentral gyrus**	**0.2532**	**0.9000**	**0.6468**	**0.0071**	**-0.0155**	**-0.0267**
R Postcentral gyrus	L Rostral anterior cingulate cortex	0.5159	0.5925	0.0766	0.0127	-0.0093	0.0099
**R Postcentral gyrus**	**L Insula**	**0.5503**	**0.6766**	**0.1263**	**0.0042**	**-0.0018**	**0.0499**
**R Postcentral gyrus**	**R Banks superior temporal sulcus**	**0.7046**	**0.7122**	**0.0077**	**0.0016**	**-0.0050**	**0.0592**
**R Postcentral gyrus**	**R Caudal middle frontal cortex**	**0.5370**	**0.7685**	**0.2315**	**0.0011**	**-0.0261**	**-0.0977**
**R Postcentral gyrus**	**R Middle temporal cortex**	**0.4660**	**0.8269**	**0.3609**	**0.0016**	**-0.0089**	**0.0312**
**R Postcentral gyrus**	**R Paracentral gyrus**	**0.5012**	**0.9072**	**0.4060**	**0**	**-0.0104**	**-0.0086**
R Posterior cingulate cortex	***L Middle temporal cortex***	***0.6617***	***0.6765***	***0.0148***	***0.0011***	***0.0022***	***0.0300***
**R Posterior cingulate cortex**	**R Caudal middle frontal cortex**	**0.5351**	**0.7375**	**0.2023**	**0.0096**	**0.0022**	**-0.0004**
R Precentral gyrus	L Inferior temporal cortex	0.3852	0.7467	0.3616	0.0370	-0.0007	0.0280
R Precentral gyrus	L Middle temporal cortex	0.4119	0.7418	0.3299	0.0127	-0.0011	0.0118
**R Precentral gyrus**	**L Insula**	**0.5031**	**0.6990**	**0.1959**	**0.0090**	**-0.0044**	**0.0169**
**R Precentral gyrus**	**R Caudal middle frontal cortex**	**0.3796**	**0.8449**	**0.4654**	**0**	**-0.0040**	**-0.0262**
R Precuneus	L Middle temporal cortex	0.5734	0.7451	0.1717	0.0208	-0.0015	0.0229
R Rostral anterior cingulate cortex	*L Cuneus*	*0.6758*	*0.4519*	*-0.2239*	*0.0260*	*0.0159*	*0.0270*

Bolton, T., Van De Ville, D., Régis, J., Witjas, T., Girard, N., Levivier, M., Tuleasca, C. (2022). Supporting information for "Morphometric features of drug-resistant essential tremor and recovery after stereotactic radiosurgical thalamotomy." *Network Neuroscience*. Advance publication. https://doi.org/netn_a_00253

**R Rostral anterior cingulate cortex**	**L Isthmus cingulate**	**0.6920**	**0.4839**	**-0.2081**	**0.0094**	**0.0039**	**-0.0586**
**R Rostral middle frontal cortex**	**L Pars triangularis**	**0.5139**	**0.9360**	**0.4221**	**0.0024**	**-0.0075**	**-0.0098**
R Rostral middle frontal cortex	L Supramarginal gyrus	0.4484	0.7657	0.3173	0.0476	-0.0032	-0.0027
R Rostral middle frontal cortex	*L Insula*	*0.6901*	*0.7519*	*0.0618*	*0.0280*	*0.0017*	*0.0282*
**R Rostral middle frontal cortex**	**R Pars triangularis**	**0.4605**	**0.9190**	**0.4585**	**0.0016**	**0.0014**	**-0.0065**
R Superior frontal cortex	*L Middle temporal cortex*	*0.4273*	*0.8234*	*0.3962*	*0.0206*	*0.0005*	*0.0177*
R Superior frontal cortex	*L Supramarginal gyrus*	*0.3477*	*0.8430*	*0.4953*	*0.0206*	*0.0013*	*0.0089*
R Superior frontal cortex	L Insula	0.7259	0.7712	0.0453	0.0127	-0.0025	0.0238
R Superior parietal cortex	L Middle temporal cortex	0.6464	0.8378	0.1915	0.0208	-0.0001	0.0140
R Superior parietal cortex	L Insula	0.5867	0.7194	0.1327	0.0260	-0.0055	0.0089
**R Superior temporal cortex**	**L Middle temporal cortex**	**0.3864**	**0.8567**	**0.4703**	**0.0011**	**-0.0108**	**-0.0222**
**R Superior temporal cortex**	**L Rostral anterior cingulate cortex**	**0.6452**	**0.7240**	**0.0789**	**0.0081**	**-0.0120**	**-0.0281**
R Superior temporal cortex	L Insula	0.5390	0.6942	0.1552	0.0352	-0.0014	0.0258
R Superior temporal cortex	*R Banks superior temporal sulcus*	*0.4554*	*0.7050*	*0.2497*	*0.0223*	*0.0043*	*0.0563*
R Superior temporal cortex	R Postcentral gyrus	0.2047	0.8403	0.6356	0.0409	-0.0131	-0.0333
R Supramarginal gyrus	L Supramarginal gyrus	0.3534	0.8187	0.4653	0.0403	-0.0034	-0.0158
R Frontal pole	*L Insula*	*0.9548*	*0.6786*	*-0.2761*	*0.0250*	*0.0094*	*0.1037*
R Temporal pole	L Postcentral gyrus	0.4905	0.8049	0.3143	0.0223	-0.0322	-0.0919
**R Temporal pole**	**R Caudal middle frontal cortex**	**0.8318**	**0.7647**	**-0.0671**	**0.0011**	**-0.0253**	**-0.0853**
R Temporal pole	R Inferior temporal cortex	0.5208	0.7377	0.2169	0.0202	-0.0295	-0.0905
R Temporal pole	***R Rostral anterior cingulate cortex***	***0.7515***	***0.4768***	***-0.2747***	***0.0020***	***0.0145***	***0.0196***
R Transverse temporal cortex	***L Middle temporal cortex***	***0.6254***	***0.7156***	***0.0901***	***0.0094***	***0.0060***	***0.0325***
R Transverse temporal cortex	***R Caudal middle frontal cortex***	***0.6197***	***0.7895***	***0.1698***	***0.0057***	***0.0084***	***0.0318***
**R Insula**	**L Middle temporal cortex**	**0.6864**	**0.7153**	**0.0289**	**0.0016**	**-0.0027**	**0.0116**
R Insula	L Insula	0.4563	0.7736	0.3173	0.0223	-0.0029	0.0041
R Insula	***R Caudal middle frontal cortex***	***0.6419***	***0.8143***	***0.1724***	***0.0011***	***0.0029***	***0.0060***
R Insula	R Postcentral gyrus	0.4047	0.7990	0.3943	0.0134	-0.0236	-0.0517
L Caudate	R Supramarginal gyrus	0.7084	0.5677	-0.1408	0.0277	-0.0020	0.0225
L Putamen	L Banks superior temporal sulcus	0.8754	0.5956	-0.2798	0.0359	-0.0282	-0.0848
L Putamen	L Middle temporal cortex	0.7042	0.4888	-0.2154	0.0348	-0.0056	-0.0268
**L Putamen**	**R Middle temporal cortex**	**1.0122**	**0.6435**	**-0.3687**	**0.0069**	**0.0110**	**-0.0077**
**L Putamen**	**R Supramarginal gyrus**	**0.7777**	**0.4842**	**-0.2935**	**0.0073**	**-0.0080**	**-0.0447**
L Pallidum	L Banks superior temporal sulcus	0.8302	0.6549	-0.1753	0.0176	-0.0030	-0.0002
L Pallidum	***L Inferior temporal cortex***	***0.9568***	***0.7401***	***-0.2167***	***0.0046***	***0.0350***	***0.0815***
L Pallidum	L Lateral occipital cortex	0.7155	0.6942	-0.0213	0.0274	0.0016	-0.0542
L Pallidum	*L Middle temporal cortex*	*0.8789*	*0.5939*	*-0.2851*	*0.0159*	*0.0110*	*0.0400*
L Pallidum	L Paracentral gyrus	0.5755	0.6915	0.1160	0.0283	-0.0063	-0.0479
L Pallidum	L Precentral gyrus	0.5717	0.6259	0.0541	0.0481	-0.0013	-0.0547
L Pallidum	L Rostral anterior cingulate cortex	0.6018	0.5052	-0.0966	0.0437	-0.0081	-0.0548
L Pallidum	L Superior frontal cortex	0.6756	0.5953	-0.0804	0.0159	-0.0009	-0.0683
L Pallidum	L Superior temporal cortex	0.6922	0.6114	-0.0808	0.0136	0.0072	-0.0242
L Pallidum	*L Transverse temporal cortex*	*0.5925*	*0.6473*	*0.0549*	*0.0471*	*0.0106*	*0.0393*
L Pallidum	R Pars orbitalis	0.6942	0.6149	-0.0793	0.0223	0.0015	-0.0675
**L Pallidum**	**R Postcentral gyrus**	**0.7155**	**0.5207**	**-0.1947**	**0.0049**	**-0.0062**	**-0.0820**
L Pallidum	R Rostral middle frontal cortex	0.6919	0.4733	-0.2186	0.0125	0.0000	-0.0457
L Pallidum	R Superior frontal cortex	0.6549	0.6199	-0.0349	0.0195	0.0047	-0.0272
L Pallidum	*R Superior temporal cortex*	*0.6538*	*0.5924*	*-0.0614*	*0.0315*	*0.0207*	*0.0269*
L Pallidum	R Frontal pole	0.6550	0.4968	-0.1582	0.0280	0.0005	-0.0776

Bolton, T., Van De Ville, D., Régis, J., Witjas, T., Girard, N., Levivier, M., Tuleasca, C. (2022). Supporting information for "Morphometric features of drug-resistant essential tremor and recovery after stereotactic radiosurgical thalamotomy." *Network Neuroscience*. Advance publication. https://doi.org/netn_a_00253

L Pallidum	R temporal pole	0.6696	0.6165	-0.0532	0.0267	0.0006	-0.0417
**L Accumbens nucleus**	**L Hippocampus**	**0.9837**	**0.5402**	**-0.4435**	**0.0090**	**-0.0062**	**-0.0403**
R Caudate	L Pars triangularis	0.7987	0.3404	-0.4583	0.0238	-0.0076	-0.0329
R Caudate	L Postcentral gyrus	0.8827	0.3950	-0.4877	0.0294	-0.0067	-0.0272
R Caudate	L Precentral gyrus	0.9685	0.4772	-0.4913	0.0331	-0.0033	-0.0154
R Caudate	*L Superior temporal cortex*	*0.8723*	*0.3064*	*-0.5659*	*0.0208*	*0.0033*	*0.0063*
R Caudate	*R Parahippocampal gyrus*	*1.0116*	*0.3945*	*-0.6171*	*0.0206*	*0.0047*	*0.0076*
R Caudate	R Paracentral gyrus	0.8504	0.4970	-0.3535	0.0292	-0.0057	-0.0209
R Caudate	*R Precentral gyrus*	*0.9790*	*0.5314*	*-0.4476*	*0.0223*	*0.0076*	*0.0140*
R Caudate	*R Supramarginal gyrus*	*0.8433*	*0.4906*	*-0.3527*	*0.0457*	*0.0060*	*0.0143*
R Putamen	R Middle temporal cortex	0.7758	0.4768	-0.2990	0.0161	0.0014	-0.0552
**R Hippocampus**	**L Accumbens nucleus**	**1.1681**	**0.7407**	**-0.4275**	**0.0042**	**-0.0081**	**-0.0538**
R Accumbens nucleus	L Accumbens nucleus	0.6357	0.7124	0.0767	0.0286	-0.0200	-0.0904
Brainstem	R Cerebellum white matter	0.7297	0.6632	-0.0665	0.0491	-0.0137	-0.0990
